# Psychological predictors of negative treatment outcome with Erenumab in chronic migraine: data from an open label long-term prospective study

**DOI:** 10.1186/s10194-021-01333-4

**Published:** 2021-10-02

**Authors:** Bottiroli Sara, Roberto De Icco, Gloria Vaghi, Stefania Pazzi, Elena Guaschino, Marta Allena, Natascia Ghiotto, Daniele Martinelli, Cristina Tassorelli, Grazia Sances

**Affiliations:** 1 Faculty of Law, Giustino Fortunato University, Benevento, Italy; 2 Headache Science and Neurorehabilitation Center, IRCCS Mondino Foundation, Pavia, Italy; 3 Department of Brain and Behavioral Sciences, University of Pavia, Pavia, Italy

**Keywords:** Anti-CGRP monoclonal antibody, Open label, Chronic migraine, Personality, Anxiety, Stressful event, Alexithymia

## Abstract

**Background:**

Monoclonal antibodies (mABs) targeting the calcitonin gene-related peptide (CGRP) pathway represent the first disease-specific preventive migraine therapy. Growing evidence suggests that they are effective in the preventive treatment of difficult-to-treat patients. In this study, we evaluated the psychological predictors of the outcome of treatment with the anti-CGRP monoclonal antibody erenumab in patients with chronic migraine (CM).

**Methods:**

Seventy-five patients with CM who had already failed at least 3 preventive therapies received erenumab every 28 days for a period of 12 months. Before the first administration, patients received a full psychological evaluation using The Structured Clinical Interview for DSM-5 Clinician Version (SCID-5-CV) to assess personality disturbances (primary outcome), mood and anxiety disorders, and as well specific questionnaires to evaluate alexithymia traits, childhood traumas, and current stressors (secondary outcomes).

**Results:**

After 12 months of treatment, 53 patients reported a reduction of at least 50% in headache days/per month (Responders), whereas 22 did not (Non Responders). When compared to Responders, Non Responders were characterized by a higher prevalence of personality disorders belonging to Cluster C (avoidant, dependent, and obsessive-compulsive) (77% vs 37%, *p* = .001). Non Responders were also characterized by a higher prevalence of anxiety disorders (90% vs 60%, *p* = 0.007), showed more alexithymic traits (51.7 ± 13.7 vs 42.9 ± 14.3, *p* = 0.017), and reported a higher number of 'at least serious' current stressors (3.2 ± 4.0 vs 0.8 ± 1.4, *p <* .0001) than Responders. At the multivariate analysis, higher prevalence of Cluster C personality disorders (OR 3.697; *p* = 0.05) and higher number of ‘at least serious’ life events (OR 1.382; *p* = 0.017) arose as prognostic factors of erenumab failure.

**Conclusions:**

Erenumab confirmed its effectiveness in a population of difficult-to-treat migraine. The presence of “anxious-fearful” personality together with current stressors and anxiety represent negative predictors of treatment outcome.

**Trial registration:**

The study protocol was registered at clinicaltrials.gov (NCT04361721).

## Background

Chronic migraine (CM), psychological disturbances, and acute medication overuse (MO) are closely linked in the clinical setting. CM and CM + MO are indeed associated with anxiety, depression, and personality disorders [1–6]. An association has also been detected between CM + MO and childhood traumas, current stressful events, and alexithymia [7, 8], conditions that are likely to play a role in the outcome of a detoxification treatment [2]. Hence, the investigation of all these psychological disturbances in CM/CM + MO becomes very important given they might affect the course of the disease itself as well as the response to treatment [9].

In the field of treatment, many options are available, including various classes of medications originally developed for other conditions and used as preventive therapy for migraine [10]. The therapeutic armamentarium for migraine prevention has recently benefitted from the arrival of the monoclonal antibodies (mABs) targeting the calcitonin gene-related peptide (CGRP) pathway [11]. These represent the first disease-specific preventive migraine therapy. CGRP is a potent vasodilator and neuropeptide in the trigeminovascular system, whose activation seems to be involved in pain and in other migraine symptoms [12]. Growing evidence suggests that mABs targeting CGRP are effective in the preventive treatment of difficult-to-treat patients such as those who had previously failed multiple prevention treatments [13] or those with MO [14]. Interestingly, from a psychological and clinical point of view, these “difficult-to-treat” patients are particularly challenging being characterized by the presence of a high number of psychiatric comorbidities and personality disorders with respect to those that respond to therapies [3, 6]. The presence of psychological comorbidities is indeed associated with a worse clinical condition, development of MO, and reduced efficacy of pharmacological preventive therapies [15, 16]. Psychiatric comorbidities such as personality and mood disorders are known to have an impact on treatment effectiveness in difficult-to-treat CM/CM + MO [4, 5, 17–19]. A growing area of research pertains early life traumas and stressful events in these patients. These psychosocial variables seem to be capable to increase headache-related features, such as frequency, severity, and chronicity (e.g., [20, 21]) and can have a negative impact on the outcome of treatment in case of MO [2]. This is because, according to the bio-psychosocial model, there is a composite interaction between psychological, psychosocial, and biological aspects, reciprocally influencing each other [22]. To the best of our knowledge, no study has so far evaluated detailed psychological variables associated to the success/failure of a treatment with an anti-CGRP monoclonal antibody in CM patients.

Keeping this in mind, the aim of this study was the identification of psychological variables that may be predictive of the long-term outcome of the treatment with the anti-CGRP monoclonal antibody erenumab. The role of personality disorders in predicting treatment response was considered as the primary outcome measure of this study; the role of mood and anxiety disorders, childhood traumas, stressful events, and alexithymic traits was evaluated as a secondary outcome. The working hypothesis is that patients not responding to this new preventive treatment bear a more complex psychological profile than those responding. This is an unexplored area of clinical research that merits further attention given that the identification of such predicting factors would hopefully prompt optimization of the management.

## Patients and methods

This study has been conducted from 2020 to 2021 at the Headache Science and Neurorehabilitation Center (a tertiary referral center) of the Mondino Foundation in Pavia, Italy. The study was approved by the local Ethics Committee and registered in https://clinicaltrials.gov (NCT04361721).

### CM patients

Inclusion criteria were: (a) age > 18, < 65 years, (b) fulfillment of ICHD-3 criteria [23] for CM or CM + MO for at least 12 months prior to enrollment, (c) previous failure of at least three different pharmacological classes of preventive therapies. Exclusion criteria were: (a) dementia, (b) previous diagnosis of psychosis, and (c) mental retardation. A previous therapeutic failure was defined as: a) no reduction (< 30%) in headache frequency after at least 6 weeks of treatment with an adequate dose, or b) the subject discontinued the treatment due to related adverse events or poor tolerability. An expert neurologist verified the eligibility criteria during the recruitment process based on history, headache diaries, and neurological evaluation.

### Procedure

All patients underwent a screening visit during which they signed a written informed consent after a thorough description of the protocol by the investigator. Patients who fulfilled inclusion/exclusion criteria were enrolled in the study and underwent a one-month baseline observation period (BP) aimed to prospectively confirm headache frequency as well as the diagnosis of CM or CM + MO. At the end of the BP, patients underwent a psychological evaluation (clinical interview and questionnaires) and then received the first injection of erenumab (70 mg). The treatment was repeated every 28 days with the possibility to increase the dose to 140 mg, based on clinical judgment, for a total of 13 doses delivered over a period of 12 months. During the entire treatment period, patients recorded prospectively headache characteristics, use of drugs, and possible side effects on an ad hoc diary. Patients were seen regularly, at least quarterly, at the Center. At the end of the one-year period, the treatment was discontinued in all patients for at least 3 months, and a clinical follow up was planned. More information about the procedures is reported in [24].

### Psychological evaluation

CM patients underwent a complete psychological evaluation performed by two expert psychologists based on Diagnostic and Statistical Manual of Mental Disorders (DSM-5) criteria [25] by using the Structured Clinical Interview for DSM-5, Clinical Version (SCID-5-CV) [26] for assessing personality disorders as well as mood and anxiety disturbances. Interview questions were provided alongside each DSM-5 criterion to aid users in rating each criterion as either present or absent. Personality disorders comprise 10 disorders, which can be grouped into Cluster A (paranoid, schizoid, and schizotypal), Cluster B (antisocial, borderline, histrionic, and narcissistic), and Cluster C (avoidant, borderline, and dependent) according to the shared characteristics. Anxiety disorders include specific phobias, social anxiety disorder, and generalized anxiety disorder, as well as panic disorder and agoraphobia. Mood disorders include bipolar disorder, cyclothymia, major depressive disorder, disruptive mood dysregulation disorder, persistent depressive disorder, and premenstrual dysphoric disorder.

Participants also filled a series of questionnaires. The Italian version of the Hospital Anxiety and Depression Scale (HADS) [27] was used to assess anxiety and depression symptomatology. This questionnaire comprises seven items concerning depression and seven items for anxiety, graded on a four-point (0–3) Likert scale, so that possible scores ranged from 0 to 21 for both depression and anxiety.

The Italian version (adapted by [8]) of the Childhood Trauma Questionnaire was used to assess childhood traumas. For each item, patients were requested to indicate whether they experienced each kind of trauma and its impact on a 5-points Likert scale ranging from mild to very serious. This version of the scale, as evaluated in the present study, has good internal consistency (Cronbach’s alpha = 0.76). The Italian version [8] of the Stressful Life-events Questionnaire was used to assess current stressful life events (e.g., moving, divorce, new work, dismissal, etc.) and has a good internal consistency (Cronbach’s alpha = 0.85), as resulted from our data. For each item, patients were requested to indicate whether they recently experienced each kind of stressful life event and its impact on a 5-points Likert scale ranging from mild to very serious. For both the Childhood Trauma Questionnaire and the Stressful Life-events Questionnaire, we considered the total number of traumas/life-events reported and we distinguished them according to their level of impact. A further index derived from evaluating the number of events with ‘at least a serious' level of impact was considered, which was derived by summing the serious and very serious impacts within each questionnaire.

The 20-item Italian version [28] of the Toronto Alexithymia Scale (TAS-20) was used to evaluate the presence of alexithymia. Items in the first factor (Factor 1) were referred to the ability to identify feelings and distinguish them from bodily sensations. Items in the second factor (Factor 2) related to a concrete thinking style. Items in the third factor (Factor 3) concerned the ability to express emotion and fantasy.

### Definition of treatment outcome

Erenumab treatment was considered successful when the number of monthly migraine headache days in the last month of the treatment period was reduced by at least of 50% with respect to the BP; whereas it was considered ineffective when the reduction in migraine frequency was < 50%. Based on the above cut-off, patients were subdivided into Responders and Non Responders. The demographic and clinical characteristics of these two groups are reported in Table 1.

**Table 1:** Baseline clinical characteristics of study population. Data are presented as “mean ± standard deviation” or “absolute value (percentage)”

	Total ***n*** = 75	Responders ***n*** = 53	Non Responders ***n*** = 22	***p***
Age	49.5 ± 9.4	49.4 ± 9.4	49.6 ± 9.6	0.93
Gender, female	53 (71%)	35 (66%)	18 (82%)	0.14
Age at onset (year)	14.7 ± 7.0	14.9 ± 7.2	14.2 ± 6.6	0.67
Duration of chronic migraine (years)	12.2 ± 9.3	12.4 ± 9.1	11.7 ± 8.0	0.78
CM	5 (7%)	4 (7%)	1 (5%)	0.54
CM + MO	70 (93%)	49 (93%)	21 (95%)	0.67
Migraine days per month	22.7 ± 5.1	22.8 ± 4.8	22.4 ± 5.7	0.73
Headache days per month	24.3 ± 4.9	24.2 ± 4.9	24.5 ± 4.9	0.79
Days of acute drug intake per month	20.5 ± 7.4	20.5 ± 7.6	20.5 ± 7.1	0.99
Acute treatment				0.74
NSAIDs	13 (18%)	10 (19%)	3 (14%)	
Triptans	18 (24%)	14 (26%)	4 (18%)	
Combination-analgesic drug	39 (52%)	26 (49%)	13 (59%)	
Multiple drug classes	5 (8%)	3 (6%)	2 (9%)	
Patients on preventive treatment at BP	41 (55%)	30 (57%)	11 (50%)	0.39
Patients with other pain conditions	8 (10%)	7 (13%)	1 (5%)	0.42
Average pain severity at BP (NRS score, range 0–10)	7.4 ± 1.3	7.2 ± 1.3	7.6 ± 1.2	0.15
No. of previously failed preventive treatments	4.0 ± 1.3	3.9 ± 1.4	4.1 ± 1.1	0.65

*CM* chronic migraine, *CM + MO* chronic migraine associated to medication overuse, *NSAIDs* nonsteroidal anti-inflammatory drugs, *Combination-analgesic drug* formulation combining drugs of two or more classes, each with analgesic effect or acting as adjuvants, *BP* baseline observation period, *NRS* Numerical Rating Scale

**Table 2:** Psychological characteristics of the two groups as result from the clinical interview based on SCID-5-CV

	Responders n (%)	Non Responders n (%)	*p*
**Personality disorders**	**21 (40%)**	**18 (82%)**	**0.001**
*Cluster A*	1 (2%)	0 (0%)	0.70
Paranoid	1 (2%)	1 (5%)	0.50
Schizoid	0 (0%)	0 (0%)	–
Schizotypal	0 (0%)	0 (0%)	–
*Cluster B*	6 (12%)	4 (18%)	0.34
Histrionic	2 (4%)	0 (0%)	0.49
Narcissistic	2 (4%)	1 (5%)	0.65
Antisocial	0 (0%)	0 (0%)	–
Borderline	3 (6%)	3 (14%)	0.23
***Cluster C***	**19 (37%)**	**17 (77%)**	**0.001**
Avoidant	5 (9%)	5 (23%)	0.12
Dependent	4 (8%)	3 (14%)	0.33
**Obsessive-compulsive**	**19 (36%)**	**15 (68%)**	**0.01**
Mood disorders	26 (49%)	11 (50%)	0.57
**Anxiety disorders**	**32 (60%)**	**20 (90%)**	**0.007**

Significant differences are bolded. *SCID-5-CV* The Structured Clinical Interview for DSM-5 Clinician Version

### Primary and secondary outcome measures

The data reported in the present study pertain to the secondary analysis from an open-label study on erenumab in CM, whose primary outcome was the evaluation of sensitization [24]. In the context of the present study, the frequency at baseline of personality disorders, detected using the SCID-5-CV interview, was considered as primary-outcome measure. Frequency of mood and anxiety disorders, the number of childhood traumas and stressful life events, and alexithymic traits, detected with the SCID-5-CV interview and questionnaires, at baseline were evaluated as secondary outcome measures.

### Statistical procedures

Data were presented as mean ± standard deviation for continuous data and as n/% for frequency data. The differences between Responders and Non Responders were examined with χ^2^ tests for categorical variables and one-way analysis of variance (ANOVA) for quantitative variables. Multivariate logistic regressions (enter method) were applied. The criterion for variables inclusion in the multivariate model was the existence of significant differences among groups in the ANOVAs. An alpha of 0.05 was used for all statistical tests. All analyses were conducted using SPSS (Statistical Package for Social Sciences, version 23.0).

The sample size was calculated on the primary outcome measure. Based on [29] showing a prevalence of 81% of personality disorders in CM, it was expected a prevalence of 60% in Responders and of 95% in Non Responders. Hence, a minimum sample size of 22 patients per group (Responders and Non Responders) would be sufficient for 95% confidence interval (two-sided) and 80% power.

## Results

### Patient population

**Fig. 1:**
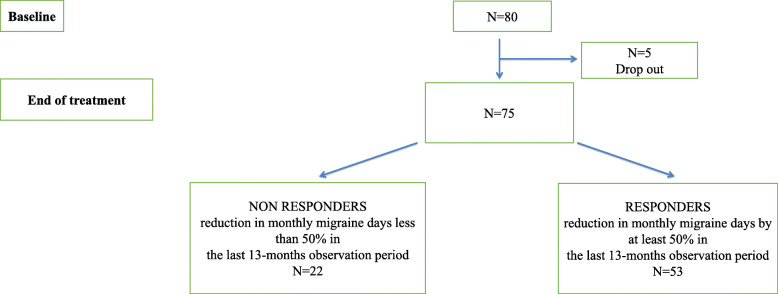
Clinical outcome of the treatment period

As represented in Figs. 1, 80 patients were recruited for this study and 75 completed the 13-dose treatment period (71% females; mean age 49.5; age range 22–65). The reason for dropping out were consent withdrawal due to self-experienced treatment “failure”. A total of 71 patients were switched from the 70-mg dose to the 140-mg dose after the initial 70-mg dose. Considering the final sample, 53 patients (71%) (66% females, mean age: 49.4; age range 22–65) reported a > 50% reduction in the number of monthly migraine days with respect to baseline (Responders) and 22 patients (29%) (82% females; mean age 49.6; age range 28–61) did not (Non Responders) (Fig. 1).

### Comparison between non responders and responders

When comparing demographic and clinical features between Non Responders and Responders, as evident from Table 1, no significant differences were found.

#### Primary outcome measure

At the clinical interview based on SCID-5-CV evaluation (Table 2), some differences resulted between Non Responders and Responders as regards personality profiles. The Non Responder group had a higher prevalence of personality disorders (*χ*^2^ (1, *N* = 75) = 11.09, *p* = 0.01). More in detail, the Non Responder group was more prevalently characterized by patients showing obsessive-compulsive personality disorder (*χ*^2^ (1, *N* = 75) = 6.56, *p* = 0.01) and personality disorders belonging to Cluster C (*χ*^2^ (1, *N* = 75) = 10.27, *p* = 0.001) than the Responder group. No other differences resulted between groups.

#### Secondary outcome measures

As regards the other psychological variables assessed via the SCID-5-CV, the Non Responder group showed a higher prevalence of anxiety disorders (*χ*^2^ (1, *N* = 75) = 6.82, *p* = 0.007) than the Responder group. No other differences resulted between the two groups from the clinical interview.

When considering psychological variables measured via questionnaires (Table 3), some differences resulted between Non Responders and Responders in the Stressful Life-events Questionnaire. Indeed the Non Responder group reported a higher number of stressful events in the past (*F*(1, 73) = 4.22, *p* = 0.044), in particular of those with serious (*F*(1, 73) = 6.64, *p* = 0.012), very serious (*F*(1, 73) = 14.17, *p* < 0.001), and ‘at least serious’ (*F*(1, 73) = 15.64, *p* < 0.001) impact than the Responder group. The same trend was found for the TAS-20 total score (*F*(1, 73) = 5.98, *p* = 0.017) and TAS-20 Factor 1 (*F*(1, 73) = 6.86, *p* = 0.011), with the Non Responder group scoring higher than the Responder one. No other differences resulted between these two groups in the other questionnaires.

**Table 3:** Psychological characteristics of the two groups as result from self-reported questionnaires at the baseline evaluation. Data are presented as “mean ± standard deviation”

	Responders N = 53	Non Responders N = 22	*p*
HADS Depression	6.1 ± 4.6	7.5 ± 5.3	0.28
HADS Anxiety	6.1 ± 4.1	7.1 ± 4.1	0.35
Childhood Trauma Questionnaire			
Important traumas	0.4 ± 0.8	0.5 ± 1.3	0.65
Serious traumas	0.1 ± 0.4	0.3 ± 0.6	0.21
Very serious traumas	0.3 ± 0.7	0.4 ± 1.0	0.62
At least serious traumas	0.4 ± 0.9	0.6 ± 1.6	0.38
Total traumas	1.3 ± 1.5	1.4 ± 2.4	0.83
Stressful life-events Questionnaire			
Important events	2.5 ± 2.7	3.2 ± 4.7	0.41
**Serious events**	**0.5 ± 1.1**	**1.4 ± 1.8**	**0.012**
**Very serious events**	**0.3 ± 0.7**	**1.9 ± 2.9**	**< 0.001**
**‘At least serious’ events**	**0.8 ± 1.4**	**3.2 ± 4.0**	**< 0.001**
**Total stressful events**	**7.1 ± 5.2**	**10.7 ± 9.8**	**0.044**
TAS-20			
**Total score**	**42.9 ± 14.3**	**51.7 ± 13.7**	**0.017**
**Factor 1**	**14.2 ± 7.6**	**19.1 ± 7.0**	**0.011**
Factor 2	11.3 ± 5.0	13.1 ± 4.9	0.17
Factor 3	17.4 ± 5.4	19.6 ± 6.8	0.15

*HADS* Hospital Anxiety and Depression Scale, *TAS-20* Toronto Alexithymia Scale. Significant differences are bolded

### Psychological predictors of erenumab failure

Due to the strong associations existing between variables that were statistically significant in the previous analyses, only those variables considered as more representative for each of the investigated constructs were included in the logistic regression models. In order to further evaluate the association between personality disorders and erenumab failure, two logistic regression models were carried out, that is, one considering the prevalence of Cluster C personality disorders as covariate (Table 4) and the other considering the obsessive-compulsive personality disorder as covariate (Table 5). The rational for carrying out these two separate models was to explore the impact of personality disorders treated globally as Cluster C as well as the specific value of the obsessive-compulsive personality disorder.

**Table 4:** Model fit of logistic regression equations to predict erenumab failure (including Cluster C personality disorders)

	Multivariate OR	95% CI	***p*** value
SCID-5-CV			
**Cluster C personality disorders (yes vs no)**	**3.697**	**1.001–13.656**	**0.05**
**Anxiety disorders (yes vs no)**	4.416	0.619–31.489	0.14
Stressful life-events Questionnaire			
**Total number of ‘at least serious’ stressful events**	**1.382**	**1.061–1.801**	**0.017**
TAS-20			
**Total score**	1.027	0.982–1.074	0.24

Significant OR are in bold. *SCID-5-CV* The Structured Clinical Interview for DSM-5 Clinician Version, *TAS-20* Toronto Alexithymia Scale

**Table 5:** Model fit of logistic regression equations to predict erenumab failure (including Obsessive-compulsive personality disorder)

	Multivariate OR	95% CI	***p*** value
SCID-5-CV			
**Obsessive-compulsive personality disorder (yes vs no)**	**4.410**	**1.169–16.632**	**0.028**
**Anxiety disorders (yes vs no)**	4.408	0.617–31.471	0.14
Stressful life-events Questionnaire			
**Total number of ‘at least serious’ stressful events**	**1.493**	**1.121–1.989**	**0.006**
TAS-20			
**Total score**	1.027	0.982–1.075	0.24

Significant OR are in bold. *SCID-5-CV* The Structured Clinical Interview for DSM-5 Clinician Version, *TAS-20* Toronto Alexithymia Scale

In the first case, in the multivariate analysis, the factors that emerged as predictor of failure to erenumab treatment were: higher prevalence of Cluster C personality disorders (OR 3.697; 95% CI 1.001–13.656, *p* = 0.05) and higher number of ‘at least serious life events’ (OR 1.382; 95% CI 1.061–1.801, *p* = 0.017). This logistic regression model was statistically significant (*χ*^*2*^(4) = 24.66, *p* < .001) and it explained 40.3% (Nagelkerke R^2^) of the variance of erenumab failure after 13 doses and correctly classified 78.4% of cases.

When considering the obsessive-compulsive personality disorder, according to the results of the multivariate analysis, the factors that emerged as predictor of failure to erenumab were: higher prevalence of obsessive-compulsive personality disorder (OR 4.410; 95% CI 1.169–16.632, *p* = 0.028) and higher number of ‘at least serious’ life events (OR 1.493; 95% CI 1.121–1.989, *p* = 0.06). This logistic regression model was statistically significant (*χ*^*2*^(4) = 26.44, *p* < .001) and it explained 42.3% (Nagelkerke R^2^) of the variance of erenumab failure and correctly classified 77.3% of cases.

## Discussion

### Overview

The results of the present study, even if preliminary due to the small sample size, showed that CM patients who respond or do not respond to a-12 month treatment with the anti-CGRP monoclonal antibody erenumab bear a different psychological burden. In detail, the presence of personality disturbances, in particular those belonging to Cluster C (including obsessive-compulsive personality disorder) and anxiety disorders together with current stressful events of serious and very serious impact, and alexithymic traits resulted as substantial determinants of failure of treatment with erenumab in CM.

### Personality profiles

A personality trait can be considered as a pervasive and stable over time pattern of acting and interpreting one’s environment, as well as oneself [30]. Many studies explored the prevalence of personality disorders in CM/CM + MO (e.g., [5, 6, 30–32]), recognizing them as factors able to complicate and interfere with headache treatment [4, 5, 33]. In particular, Cluster C reflects an “anxious-fearful” and stress reactive personality [34]. It includes people viewing the world as hostile and potentially harmful to them (i.e. avoidant), those considering themselves helpless and believing they need to attach themselves to a strong caretaker in order to get through daily life (i.e., dependent), and those having strong traits and strategies of control and responsibility (i.e., obsessive-compulsive). Interestingly, even not particularly surprising being in line with previous literature on CM (e.g., [29, 31, 35]), obsessive-compulsive personality disorder was found to be the most prevalent among Non Responders. A recent study showed that the severity of obsessive-compulsive personality disorder is closely associated with intolerance of uncertainty [36], that is defined as “a dispositional characteristic that results from a set of negative beliefs about uncertainty and its implications and involves the tendency to react negatively on an emotional, cognitive, and behavioral level to uncertain situations and events” [37]. Accordingly, the prototypical description of “obsessive personality” refers to individuals tending to adhere rigidly to their daily routine, becoming uncomfortable and anxious when something goes wrong [38, 39]. Hence, this finding needs to be interpreted also in light of the high prevalence of anxiety disorders and the high number of ‘at least serious’ stressful events and alexithymic traits characterizing Non Responders.

### Anxiety disorders and stressful life events

It is well known that CM is associated to psychiatric comorbidities, including anxiety and depression [40, 41], which may play a significant role in the way one perceives pain, copes with it, and maintains a normal lifestyle. Furthermore, evidence from other fields of research showed that adults with anxiety disorders reportedly experienced significantly more life events, perceived them as more stressful, and adapted to them less well than normal controls [42, 43]. It has been also demonstrated that childhood traumas, together with stressful events and alexithymia, are more prevalent among CM + MO with respect to patients with an episodic pattern of migraine [8] and that these factors may contribute to the outcome of the detoxification treatment [2]. Stress is the result of the inability to deal with experienced life events [44] and it is one of the most common migraine trigger factor, also implicated in migraine chronification [45, 46]. In addition, alexithymia seems to be influenced by the environmental influences, including stressful events [47]. There are indeed some pieces of evidence showing that alexithymia might also bias the perception of stress and lead to a decoupling between subjective and physiological responses to it [48, 49]. In this frame, the present findings seem to support the hypothesis of a further psychological vulnerability in these difficult-to-treat patients, which derives from the complex interaction between psychological, psychosocial, and clinical factors, in line with the bio-psychosocial model [22]. The bidirectional association between anxiety disorders and migraine, where the presence of one disorder enhances the risk for and the severity of the other, is well known [50, 51]. Even if personality disorders are usually considered maladaptive traits and behaviors stable over time, it has been demonstrated that they can change over time [52, 53]. Hence, under particular circumstances, psychological and psychosocial vulnerabilities and clinical conditions may reinforce each other and may significantly interfere with treatment outcome.

### Psychological predictors of anti-CGRP monoclonal antibody

A few other studies have sought to identify possible predictors of response to erenumab in CM. Ornello and colleagues [54] compared CM patients converting or non-converting to episodic migraine after erenumab treatment and found that depressive symptomatology was not a significant predictor of conversion. It must be noted that they selected a different timing of efficacy (months 4–6) and the numerosity of the non-converter groups was quite limited (18 subjects), which may have affected the study power. In a population of refractory CM [55], depressive and anxiety symptomatology assessed via questionnaires did not show predicting values for treatment outcome. It is however worth noting that the Authors excluded patients with comorbid personality disorders and severe psychiatric comorbidities. Furthermore, they set the response threshold to a > 30% reduction in monthly migraine days, which makes it impossible to compare the studies. In partial agreement with the present findings, a multicenter real-life study reported that the responsiveness to erenumab in high frequency migraine and CM was negatively associated with the presence of psychiatric comorbidities, based on medical records [56]. Other studies explored the psychological predictors of other classes of mABs. For instance, Smitherman et al. [57] showed that the responsiveness to galcanezumab in CM patients with comorbid anxiety and/or depression varied according to the dose administered. It must be noted that, similarly to [56], psychiatric comorbidities were simply assessed in terms of presence/absence based on medical records. Finally, Lipton et al. [58] reported that a treatment with fremanezumab resulted effective in reducing the number of headache days per month in patients with CM and comorbid depression, as assessed via a questionnaire. To date, the present study is the first to perform a thorough and detailed evaluation of psychological predictors of unfavorable long-term response to anti-CGRP monoclonal antibody in CM patients.

The neurobiological reasons behind the poorer response to erenumab observed in the CM patients with a higher burden of psychiatric diseases are not immediate. Erenumab is a large molecule that does not cross the blood brain barrier and therefore it is thought to directly act on peripheral sensitization [59, 60], even if it is not possible to totally exclude it may have additional central effects [61]. It is conceivable that our study population is characterized by a persistent central sensitization. This hypothesis is favoured by two observations: i) unmanaged stress and anxiety represent risk factors for the development of a hyperexcitable trigeminal system and central sensitization [62, 63]; and ii) the chronic exposure to migraine acute medications can further worsen central sensitization [64] and increase CGRP expression [65]. It is therefore stimulating to speculate that, in patients with CM/CM + MO and a higher emotional/psychiatric load, erenumab may indeed counteract peripheral sensitization by blocking the activation of the CGRP receptor, but this effect may be attenuated/inhibited by the simultaneous role of psychiatric comorbidities on the opposite direction. With respect to erenumab, which binds to the CGRP receptor [66], the other classes of mABs, such as galcanezumab, eptinezumab, and fremanezumab, bind to the CGRP molecule [67–69]. However, given that all these mABs target the CGRP pathway, it seems reasonable to speculate that the present findings may be generalized to the other CGRP-targeting antibodies. At variance, when considering the specific clinical population addressed in this study, namely subjects with CM/CM + MO and multiple previous prophylactic treatments failures, these results cannot be generalized to different populations. Future studies should better explore all these hypotheses.

### Implications and limitations

The present study highlighted the association between Cluster C and obsessive-compulsive personality disorders or other psychological vulnerabilities with the lack of response to anti-CGRP monoclonal antibody in CM patients. These findings are in line with the idea that specific personality/psychological disturbances are important components of the illness itself, able to influence the response to this prophylactic treatment. Hence, as patients’ management, those fitting with the profile corresponding to Non Responders in this study should be treated by clinicians with particular attention due to the high risk of treatment failure.

Some limitations in the present study suggest caution in the interpretation of results and call for further ad hoc studies. First, follow-up data about patients’ psychological state after erenumab treatment were not collected. This data would have possibly contributed interesting additional information about the potential of erenumab to modify psychological characteristics. Second, childhood trauma and current stressors were collected by means of retrospective questionnaires, which may be affected by recall bias. Third, the majority (71%) of the participants in this study were women, a distribution that is consistent with CM + MO epidemiology. However, the resulting low number of male subjects suggests caution in the interpretation and generalization of the present findings to the male sex. Fourth, though properly calculated form a statistical point of view, the sample size was relatively small, which could have limited the interpretation of our findings. This is particularly relevant when considering that many different assessments were used that may have suffered from low power. For all these reasons, future multicentric studies on larger CM populations are needed to confirm these data.

## Conclusions

The results of the present study are critical for understanding the factors that may be involved in the pathophysiology of CM and are useful for further differentiating this complex group of difficult-to-treat patients in different phenotypical and/or endotypical subtypes. Besides, these data provide useful indications as regards the need of optimizing CM management by considering patients’ psychological and psychosocial history.

## Data Availability

The datasets presented in this study can be found in online repositories. The names of the repository/repositories and accession number(s) can be found below: [Zenodo: Reservation 10.5281/zenodo.5163219].

## Abbreviations

BP: Baseline Period
CGRP: Calcitonin Gene-Related Peptide
CM: Chronic Migraine
DSM-5: Diagnostic and Statistical Manual of Mental Disorders (5th edition)
HADS: Hospital Anxiety and Depression Scale
OR: Odds Ratio
mABs: Monoclonal Antibodies
MO: Medication Overuse
NRS: Numerical Rating Scale
NSAIDs: Nonsteroidal Anti-Inflammatory Drugs
SCID-5-CV: The Structured Clinical Interview for DSM-5 Clinician Version
SPSS: Statistical Package for Social Sciences
TAS-20: Toronto Alexithymia Scale
