# Correction to: Psychological predictors of negative treatment outcome with Erenumab in chronic migraine: data from an open label long-term prospective study

**DOI:** 10.1186/s10194-021-01342-3

**Published:** 2021-11-03

**Authors:** Sara Bottiroli, Roberto De Icco, Gloria Vaghi, Stefania Pazzi, Elena Guaschino, Marta Allena, Natascia Ghiotto, Daniele Martinelli, Cristina Tassorelli, Grazia Sances

**Affiliations:** 1 Faculty of Law, Giustino Fortunato University, Benevento, Italy; 2 Headache Science and Neurorehabilitation Center, IRCCS Mondino Foundation, Pavia, Italy; 3 Department of Brain and Behavioral Sciences, University of Pavia, Pavia, Italy

**Correction to: Bottiroli The Journal of Headache and Pain (2021).**

10.1186/s10194-021-01333-4

Following the publication of the original article [1], we were notified that the Sara Bottiroli’s first and last name had been mistakenly swapped.

- Originally published name:
  Bottiroli Sara.
- Corrected name:
  Sara Bottiroli.

The original article has been corrected.
